# DNp73 improves generation efficiency of human induced pluripotent stem cells

**DOI:** 10.1186/1471-2121-13-9

**Published:** 2012-03-26

**Authors:** Yi Lin, Zuxin Cheng, Zhijian Yang, Jingui Zheng, Tongxiang Lin

**Affiliations:** 1 Stem Cell Research Center, Fujian Agriculture and Forestry University, 15 Shangxiadian Road, Cangshan District, Fuzhou 350002 Fujian, PR China; 2 Agricultural Product Quality Institute, Fujian Agriculture and Forestry University, 15 Shangxiadian Road, Cangshan District, Fuzhou 350002 Fujian, PR China

## Abstract

**Background:**

Recent studies have found that p53 and its' associated cell cycle pathways are major inhibitors of human induced pluripotent stem (iPS) cell generation. In the same family as p53 is p73, which shares sequence similarities with p53. However, p73 also has distinct properties of its own, such as two alternative promoters to express transactivation of p73 (TAp73) and N terminal deleted p73 (DNp73). Functionally, TAp73 acts similarly to p53 in tumor suppression. However, DNp73, on the other hand acts as an oncogene to suppress p53 and p73 induced apoptosis. Therefore, how can p73 have opposing roles in human iPS cell generation?

**Results:**

Transcription factors, Oct4, Sox2, Klf4 and cMyc (4TF, Yamanaka factors) are used as basal conditions to generate iPS cells. In addition, the factor of DNp73(actually alpha splicing DNp73, DNp73α) is used to generate iPS cells. The experiment found that the addition of DNp73 gene increases human iPS cell generation efficiency by 12.6 folds in comparison to human fibroblast cells transduced with only the basal conditions. Also, iPS cells generated with DNp73 expression are more resistant to *in vitro *and *in vivo *differentiation.

**Conclusions:**

This study found DNp73, a family member of p53, is also involved in the human iPS cell generation. Specifically, that the involvement of DNp73 generates iPS cells that are more resistant to *in vitro *and *in vivo *differentiation. Therefore, this data may prove to be useful in future developmental studies and cancer researches.

## Background

Human induced pluripotent stem cells hold great promise in regenerative medicine, disease modeling, and drug discovery [1,2]. However, the iPS cell generation efficiency is extremely low at around 1 from 10,000 parental cells [1,2], limiting its' use. Also, such a low efficiency suggests that major factors in de-differentiation or reprogramming have not been identified yet. Recently, a series of breakthrough discoveries have brought to attention that, blocking the important tumor suppressor protein p53, and its downstream pathways, dramatically improves generation efficiency of induced pluripotent stem cells [3-7]. The data suggest that p53 is a key link between cellular reprogramming and tumor formation since it prevents differentiated cells from transforming into pluripotent stem cells.

In 2005, we found that p53 induced differentiation of mouse embryonic stem (ES) cells by inhibiting a core transcription factor, Nanog, in the presence of stresses [8]. Nanog is a key ES cell transcription factor; Loss of Nanog expression led to rapid differentiation [9,10]. The p53 protein directly binds to the Nanog promoter to suppress its' expression level and thereby initiate ES cell differentiation into somatic like cells. Therefore, p53 acts as a transcription switch because it is able to inhibit ES cells with genetic defects to self-renew causing them to differentiate into non-stem cells, and execute high efficient apoptosis [8,9].

Previous research by Yamanaka and group reported that up to 10% transduction of p53 mouse embryonic fibroblasts (mEF) can be generatedinto iPS cells [3]. P53 gene deletion enhances efficiency about 1000-fold. In addition, p53 activity can also provide sufficient conditions to turn the terminal differentiated T lymphocytes into iPS cells. Another research team found that replacing transcription factors cMYC and KLF4 with p53 gene knockout, was enough to generate iPS cells [4]. Thus, P53 is the major inhibitor of iPS cell generation. P73 is a p53 family member with similar sequence and function as p53 [11]. However, previous studies showed that P73 has rare genetic mutation events in cancers or other conditions. In addition, the p73 gene expresses products with two alternative promoters, transactivation p73 (TAp73 and N terminal deleted p73 (DNp73) [11,12]. TAp73 functions similar to p53 in that it may play a role in cancer suppression. Conversely, DNp73 functions as an oncogene by inhibiting both p73- and p53-induced apoptosis [13].

Therefore, how can there be opposing roles in human iPS cell generation? We propose that p73 might be involved in human reprogramming. We further suggest that, DNp73 might increase the efficiency of human iPS cell generation.

## Results

### DNp73 overexpression enhanced human iPS cell generation

As control treatments, we applied conditions that were reported before by traditional four transcription factors [OCT4, SOX2, cMyc, and Klf4, referred 4TF [1,11,14]. To see if the DNp73 enhances human iPS cell generation, DNp73 was cloned by PCR from cDNA and inserted into the pMXs vector. Next, the newly generated vector was transduced with 4TF together into human fibroblast cell BJ.

At day 30, in the traditional 4TF conditions, the efficiency of iPS cell generation was at 1 from 10,000 levels from human fibroblasts BJ, similar to previous reports [1,2].

**Figure 1:**
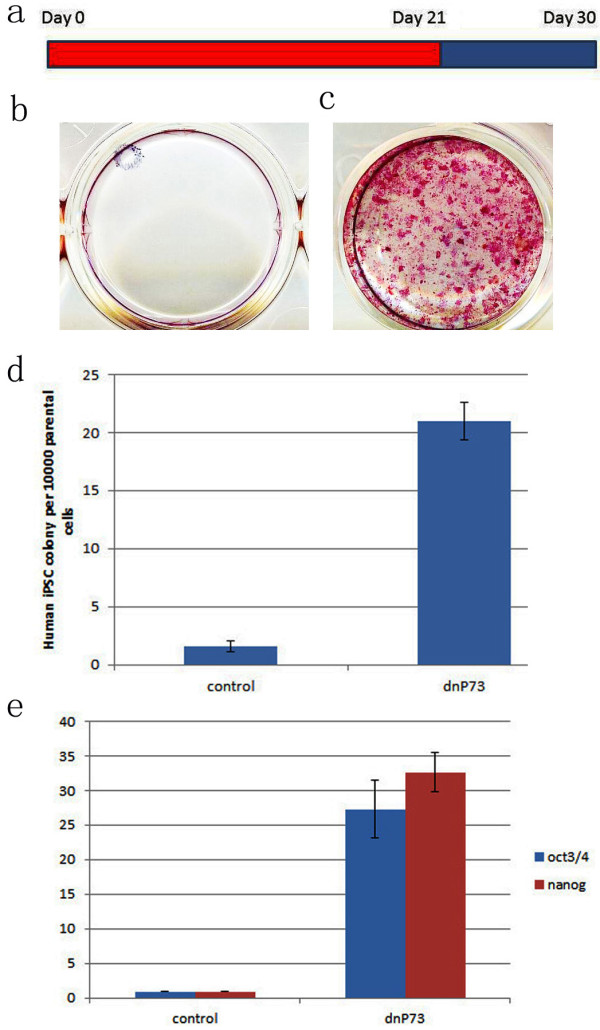
**Additional DNp73 transduction increase human iPSC generation efficiency and kinetics at day 21**. Timeline for human iPSC induction combined dnp73 withtraditional 4TF. Treatment with additional factor dnp73: culture plate had colony growth at d21 with Nanog expression and at d30 with the traditional iPS cells. a. Culture derived from 4 TF only, had only a few colonies stained by ALP+. b. While, in the culture derived from transduction of 4TF plus DNp73, many colonies appeared when stained with ALP positive at day 21. c. At day 30, large colonies seen with Nanog expression, with approx. 20 fold increase by comparison between 4TF only and 4TF plus DNp73. d. significant enhancement of gene expression oct4 (/) and Nanog (/) by Qautitative PCR detection of the cultures at d30.

In sharp contrast, at day 21 after infection, the fully reprogrammed ES like iPS cell colonies generated in 4TF plus DNp73 infected cultures yielded 21.0 colonies versus control cultures which yielded 1.67 colonies per 10,000 parental cell seeded. This translates into a 12.6 fold enhancement by the reprogrammed ES like iPS cells (Figure 1a, b, c). Furthermore, the ES like iPS cells tested positive for human ES cell marker Nanog, determined by using TRA-1-81, which dramatically increased Nanog gene expression in the culture mixture (Figure 1d, e), suggesting the cell cultures were strongly enhanced toward iPS cell generation. The expression of DNp73 gene enhanced fibroblast reprogramming suggest that p73 may also play an important role in human iPS cell generation, at least when transduced with traditional 4TF in human fibroblast cells by the traditional human iPS cell generation method.

### The human iPS cells were identified with self-renewal and pluripotency

**Figure 2:**
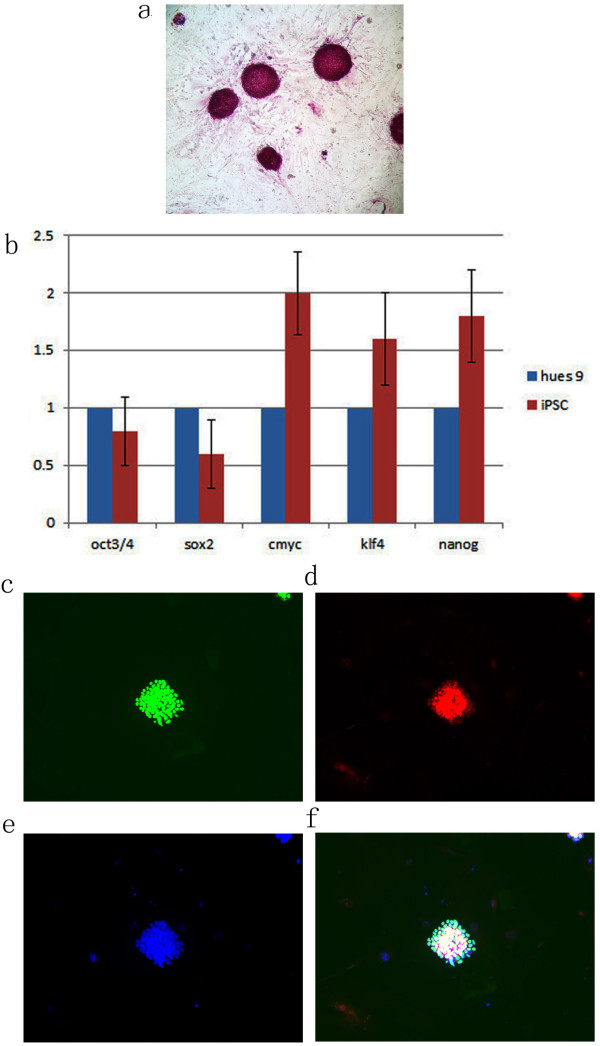
**Characterization of the passaged culture (p5) of the additional dnp73 transduced human iPS cell**. a. ALP staining of the passaged colonies. b. Real time results show the gene expression levelthat iPSC are similar to those in human ES cell. c. A typical colony of passaged cell stainied with Oct4 antibody. d. Stained with Nanog. e. DAPI cell nuclear staining. f. Merged triple stainings of c d & e.

To test the pluripotent stem cell property of self-renewal, the iPSC cells were passaged. After 12 passages, the iPS cells did not have morphological changes when their properties were characterized. Cell morphology was examined with ALP staining (Figure 2a) and real time PCR gene expression. Real time PCR results showed that these cells are similar to human embryonic stem cells huES9, which have the same or similar gene expression levels (Figure 2b). The results show that immunohistochemical staining results of the iPS cells were similar to human ES cells, with strong expressed OCT3/4 and Nanog (Figure 2c, d, e, f).

**Figure 3:**
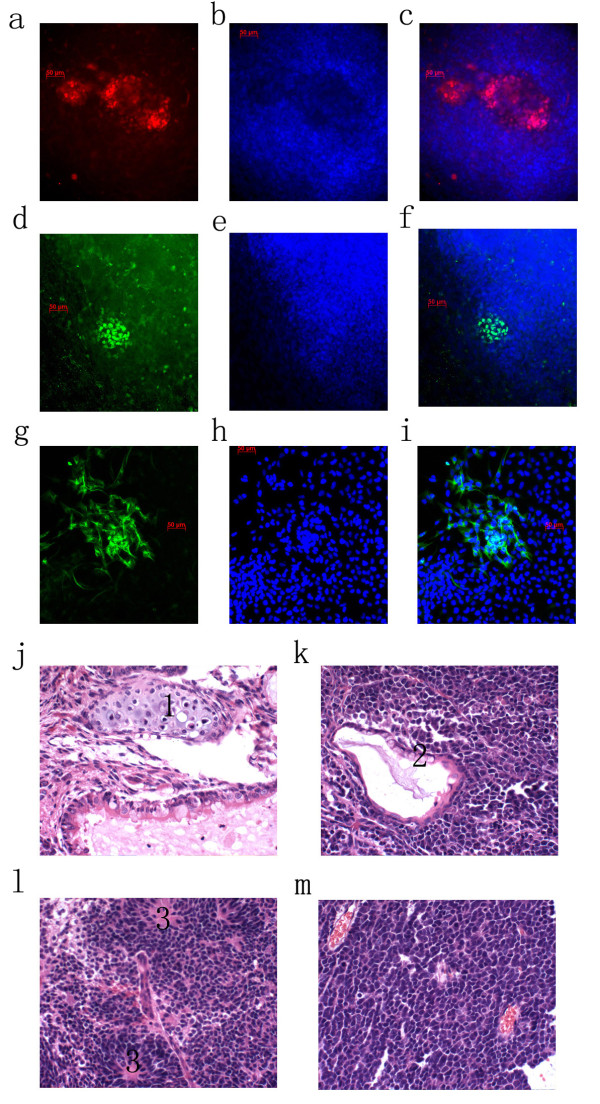
***In vitro *and *in vivo *differentiation of iPSCs generated with 4TF plus DNp73**. *In vitro *differentiation through embryoid bodies, subsequently differentiate to mesoderm (a. bracchyury staining, b. DAPI staining, c. merged), to endoderm (d. PDX1, e. DAPI, f. merged), and ectoderm (g. Tuj1 staining, h. DAPI, and i. merged). Histological sections from teratomas generated in nude mice from iPSCs consist of tissues from all three germ layers stained with hematoxylin and eosin (HE). Magnification folds 400×, J. mesoderm (1. cartilage); k. endoderm (2. goblet cells); l. ectoderm (3. neural epithelium); m. non-differentiation cells.

To test *in vitro *differentiation potential, an embryonic body mediated differentiation of iPS cells experimental methods was applied. The results showed that these iPS cells can be differentiated into derivatives of all three germ layers, including mesoderm, endoderm and ectoderm (Figure 3). These cells are Brachyury positive, a conserved role in mesoderm differentiation, and establish the embryonic mesodermal progenitor (Figure 3a-c). The endoderm cells are marked by early expression of the gene Pdx1 (Figure 3d-f). The immunocytochemistry also showed cells positive for TUJ1 stained ectoderm cells (Figure 3g-i).

To test pluripotency *in vivo*, human iPS cells were transplanted by injection into the kidney of immunodeficiency (SCID) mice. After 6 weeks, histological sections from teratomas generated in nude mice from iPS cells consisted of tissues from all three germ layers stained with hematoxylin and eosin (HE). Using 400× magnification, three germ layers of cell lines were identified, including mesoderm (cartilage) (Figure 3j), endoderm (goblet cells) (Figure 3k); ectoderm (neural epithelium) (Figure 3l).

### Resistance from differentiation cues was found in the human iPS cells generated with DNP73

We observed iPS cell differentiation under microscope at objective fields of 100× or 200×. Each sample of teratoma was randomly observed for 10 microscopic fields. We found that the iPS cell with DNp73 was resistant to *in vivo *differentiation (teratoma), which contained all undifferentiated cells in some microscopic fields (Figure 3m). We compared the 2 types of teratoma by randomly picking 10 microscopic fields. In control cells, all (100%) the microscopic fields had evidence of specific cells. However, the DNp73 derived teratoma had fewer differentiated cells, with only 20% of the fields containing all non-specific cells(Table 1)and the majority of the sections contained non-differentiated cells, 30-100% ratio. On the contrary, less than 30% non-specific cells were found in the teratoma derived from control iPSC (Table 1).

**Table 1:** Comparison observation between teratoma derived from DNp73 overexpression iPSC and those derived from control conditions of 4TF only iPSC

The iPSC for the teratoma derived	Ratio of non- differentiated cells	In 10 observed fields, the field numbers which are all non-differentiated cells
4TF teratoma	0-30%	0

4TF plus DNp73 teratoma	30-100%	3

*In vitro *differentiation demonstrated a strong difference between the cells derived from 4TF only iPS cells and those derived from iPS cells with additionally transduced DNp73. Neuronal marker cells stained by TUJ 1 antibody were observed from all 10 of the tested fields in 4TF transduced cells. However, 2 out of 10 fields stained negative in those derived from iPS cell with 4 TF plus DNp73. Furthermore, we did not find any typical bipolar TUJ1 strong expression neuronal cells. The data suggest that the DNp73 iPS cells are strongly resistant to neuronal differentiation.

### DNp73 increased ES cell core transcription factor Nanog expression

**Figure 4:**
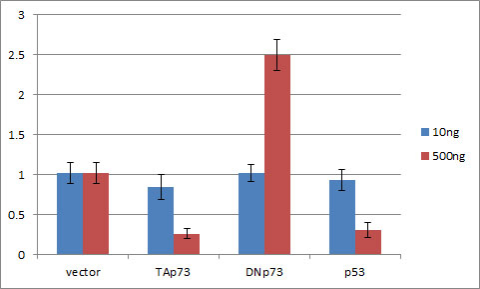
**DNp73 over expression dramatically increases Nanog promoter driven luciferase gene expression level**. While, on the contrary, p53 and TAp73 over expression significantly suppress Nanog promoter driven luciferase gene expression. The transfection construct amounts were applied at 10 ng or 500 ng per well of 24 well plates respectively. The p53 vector was served as positive control and the results were compared to treatment with vector only. Error bars: s.d. n = 3 independent experiments.

To find out how the p73 gene can improve the efficiency of iPS cells, we examined luciferase expression in mouse ES cells (Figure 4). Using our previous experimental system [8], the exogenous expression of p53 in mouse ES cells resulted in significant inhibition of the Nanog promoter-driven luciferase gene expression. We carried out TAp73 expression in the same cells, and found results similar to those from p53 transfected cells (Figure 4). However, in sharp contrast, DNp73 significantly increased Nanog gene promoter-driven luciferase levels (Figure 4). This data suggests that DNp73 directly contributes to the Nanog gene expression; thereby DNp73 has greatly improved the efficiency of pluripotent stem cells induction. Nanog is a key ES cell transcription factor. Its over expression also might enhances and improves other core ES cell transcription factors such as OCT4 expression in previous studies [15,16].

## Discussion

We found, for the first time, that additional expression of DNp73 significantly increased human iPS cell generation by 12.6 folds, and also increased the kinetics, from 30 days to 21 days. These findings suggests that p73 is an important factor involved in the generation of human iPS cells. Furthermore, p73 has a unique dual mechanism, whereby DNp73 acts as an oncogene and increases iPS cell efficiency and kinetics, while TAp73 functions similarly to p53 in tumor suppression.

The strong impact of p53 on the iPS cell generation was discovered previously [3] and Kawamura and group [4] showed that cells deficient in the p53 gene expression simplified the iPS cell generation requirement with two factors: OCT4 and SOX2. In this study, we found that DNp73 had a similar effect of enhancement on iPS cell generation as p53. This system can also be applied to detect whether C-terminal splicing proteins of p73 or another p53 member, such as p63, are also important for iPS cell generation or cell differentiation.

Grob et al. [13] found that DNp73 is capable of regulating the function of both TAp73 and p53 and is also strongly up-regulated by the TA isoforms and by p53, creating a feedback loop that tightly regulates the function of TAp73 and p53. In this study, our data strongly indicate that DNp73 expression decreases both p53 and TAp73, leading to an increase in Nanog gene expression, which subsequently benefits somatic cell reprogramming and enhances iPS cell differentiation resistance. The data also further suggests that DNp73 can be involved in tumorgenesis, at least teratoma-tumorgenesis. More extensive analysis of the expression pattern of DNp73 in other tumors and how the feedback loop is broken will require further studies.

Theunissen and Silva review [17] on Nanog expression findings as follows: The loss of Nanog alleles are more prone to differentiate but do not lose pluripotency per se; Nanog is transiently required for the specification of the naive pluripotent epiblast and is also essential to finalize somatic cell reprogramming during induction of pluripotency; Nanog acts as a molecular switch to turn on the naive pluripotent programme in mammalian cells. However, Nanog expression also enforces tumor genesis in some recent researches. Most recently, a study by Grad I, et al. found that NANOG pre-induction followed by OCT3/4, SOX2, MYC, and KLF4 induction resulted in tumour-inducing phenotype [18]. Moon JH et al. [19] reported that Nanog-induced dedifferentiation of p53-deficient mouse astrocytes into brain cancer stem-like cells. Those results underline the importance of a re-examination of the role of NANOG during reprogramming. Our results show that DNp73 derived iPS cells might increase Nanog expression. Consequently, the capacity of Nanog to resist differentiation can be regarded as a recapitulation of specification of somatic cell reprogramming, thus, resulting in a more teratomacarcinoma-like cell due to Nanog over expression. The results further indicate that p53 family members, including DNp73, might be key factors in reprogramming and cancer development. Therefore, our data provides a better understanding into tumour formation and cancer stem cell transformation, at least teratomatumorgenesis.

During iPS cell generation, global epigenetic modifications and chromatin changes, include histone modifications, DNA methylation, and chromatin remodeling [20]. Patterson et al. [21] found that genes normally unique to early embryos(LIN28A, LIN28B, DPPA4, and others)were not fully silenced in human iPS cell derivatives. LIN28 is an mRNA binding protein expressed in embryonic stem cells (Patterson), and is one of the key transcription factors for iPS cell generation [2]. Our results showed that DNp73 might be involved in the generation and *in vitro *differentiation of the iPS cells, suggesting, a link among epigenetic modifications, iPS cell gene expression and DNp73 in iPS cell generation and early embryonic development. However, the impact of DNp73 on epigenetic changes during iPS cell generation and derived cell lines has not been studied yet. A rapidly accumulating body of evidence suggests that there are important epigenetic differences between these two cell types, which seem to influence their tumorigenicity [22]. Therefore further studies on these epigenetic modifications will be very interesting.

With small molecules, we developed an efficient set of human-induced pluripotent stem cell(iPSC) methods that improve iPSC generation by over 200-fold and cut iPSC formation time in half [23]. Our results show TGF beta, MEK, WNT and ROCK signaling pathway inhibition or interference might strongly impact iPS cell generation efficiency and kinetics [24]. Whether one or more of these pathways are shared with DNp73 needs further investigation.

Should iPS cells be applied to clinical application for effective treatment of human diseases? The most suspicious concerns are that iPS cells might be cancer prone. More and more data suggests that iPS cells are very similar to human tumor cells in the epigenetics as well as genetic mutation accumulated during the generation process. Hong et al. study found that some p53 inactivation in iPS cells produced tumors [3]. Genetic safety is very critical for regenerative medicine. Further studies might be necessary to decipher whether the over expressed DNp73 is involved in the tumorigenesis, and how p73 functions in the normal iPS cells? It is not clear whether the p53 family members are a foe or friend in iPS cell generation and clinical application [23].

## Conclusions

DNp73 significantly increases human iPSC generation, and also increases the kinetics. Therefore, other p53 family members, such as DNp73, are also involved in human iPSC generation. Although the human iPS cell generated with extra expression of DNp73 could differentiate into some cell lines under certain conditions, these cells were more resistant to *in vitro *and *in vivo *differentiation cues.

Many other related questions, such as other p53 family members, the splicing variations, iPS cell generation efficiency mechanisms, related epigenetics and their interactions need to be studied.

## Methods

### Cell culture

Human fibroblasts BJ (neonatal foreskin) were purchased from American Typical Culture Center. Cell culture media reagents were from Invitrogen Corporation. The cells were maintained in DMEM containing 10% FBS, 1× MEM Non-Essential amino acid, 1× glutamax, 10 mM Hepes and 0.11 mM 2-mercaptoethanol, with/without 1× Penicillin/streptomycin. The cell was passaged 1:3-5 using 0.05% (1×) trypsin-EDTA, and re-seeded on gelatin coated plates.

### Plasmids

The pMXs vector containing the human cDNAs for OCT4 (POU5F1), SOX2, c-MYC (MYC) and KLF4, described before [1], and were obtained from Addgene. DNp73 was PCR amplified from human ES cell total cDNA and inserted into pMXs. Mouse Slc7a1 ORF was cloned into pWPXLD (Addgene), as described previously [1].

### The retroviral infection and iPS cell generation

Lentiviruse carrying Slc7a1 was produced as described before [1,8]. To improve infection efficiency, we performed triplicates of the transduction on the parental human fibroblast and checked the cells by infection with GFP expression retrovirus. A transduction was determined to be good if over 60% of the GFP expression cells produced the green fluorescence at day 3 after infection with the GFP virus.

For retrovirus production, PLAT-E packaging cells were plated at 1 × 106 cells per well in a 6-well plate. After 24 h, the cells were transfected with OCT4 (POU5F1), SOX2, KLF4, cMYC as well as DNp73. The vectors were transfected by using Fugene 6 transfection reagent (Roche) according to manufacturer's instructions. Twentyfour hours after transfection, the medium was replaced with fresh medium without antibiotics, and the plate was transferred to 32°C for retrovirus production. The viruses were collected at 48 h and 72 h and filtered with 0.45 um filter before infection of human fibroblast.

The Slc7a1-expressing human fibroblast cells were seeded at 1 × 105 cells per well in a 6 well plate on day 1. On day 2, 0.25 ml of each retroviral supernatant was added - 13 - to the cells and mixed with 1 ml fresh medium (without antibiotics) in the presence of 6 ug/ml polybrene. A second round of transduction was done on day 3. Then, cells were infected with a mixture of filtered virus, fresh medium and polybrene again.

Infection efficiency was estimated by fluorescence microscopy on cells transduced in parallel with GFP or RFP gene-carrying retroviruses.

Seven days after initial transduction, fibroblasts were harvested by trypsinization and re-plated at 1 × 104 cells per well of a 6-well plate coated with matrigel (1:50 dilution; BD Biosciences). Comparison was carrying out at 4 TF only and 4TF plus DNp73. The media was changed every 2 to 3 days depending on the cell density. The plates were then, either fixed and stained for ALP activity, or stained for protein markers, or the cultures were continued, and the ES markers were checked at day 14, 21 and 30.

### Alkaline phosphatase (ALP) staining

ALP staining was performed using ALP detection kit (Sigma) according to manufacturer's instructions.

### Immunocytochemistry staining

For immunocytochemistry, cells were fixed in 4% paraformaldehyde (10 min, room temperature), washed twice with phosphate buffer saline (PBS), blocked with PBS plus 5% normal donkey serum (Chemicon, or 2% BSA) and 0.1% TritonX-100 (15 min, room temperature) and then treated with primary antibodies overnight at 4°c. The primary antibodies used were antibodies to NANOG(Chemicon; 1:1,000); to OCT4 (Santa Cruz Biotech; 1:200), to SSEA 4 (Chemicon; 1:500), to Tra-1-81 (monoclonal antibody (mAb) Chemicon; 1:500), Beta III Tubulin (Covance Research Products Inc; 1:1000), to PDX 1(1:500) and to Brachyury (R&D; 1:500).

The cells were washed twice with PBS and then treated with secondary antibodies for 1 h at room temperature. The secondary antibodies used were Alexa fluor 488 labeled donkey anti-rabbit or anti-mouse IgG (Invitrogen; 1:1,000) and Alexa fluor 555 labeled donkey anti-rabbit or anti-mouse IgG(Invitrogen; 1:1,000). Nuclei were stained with 0.5 ug/ml DAPI(Sigma). Images were captured using a Nikon Eclipse TE2000-U/X-cite 120 EXFO microscope with a photometric camera.

### *In vitro *differentiation and teratoma assay

Generation of embryoid bodies and *in vitro *differentiation were performed as described above. For the teratoma assay, 3 to 5 million cells were injected under the kidney capsule of severe combined immunodeficient (SCID) mice. The animals used in this research were approved by the Fujian Agriculture and Forestry University Animal Research Committee.

### Real time-PCR

Total RNA was extracted from cells using RNAeasy minikit(Qiagen). cDNAs were synthesized according to product instructions using superscript III first strand synthesis kit (Invitrogen). PCR cycles using respective primers were performed. The sequences of the primers are described in previous research papers [1,8].

## Competing interests

The authors declare that they have no competing interests.

## Authors' contributions

YL and TL conducted the experiments. TL made the hypothesis, designed the experiments and wrote the manuscript. ZC and ZY provided assistance in some of the experiments. TL and JZ supervised the study. All authors read and approved the final manuscript.

## References

[B1] Takahashi K Tanabe K Ohnuki M Narita M Ichisaka T Tomoda K Yamanaka S Induction of pluripotent stem cells from adult human fibroblasts by defined factors Cell 2007 131 861 872 18035408 10.1016/j.cell.2007.11.019

[B2] Yu J Vodyanik MA Smuga-Otto K Antosiewicz-Bourget J Frane JL Tian S Nie J Jonsdottir GA Ruotti V Stewart R Slukvin II Thomson JA Induced pluripotent stem cell lines derived from human somatic cells Science 2007 318 1917 1920 18029452 10.1126/science.1151526

[B3] Hong H Takahashi K Ichisaka T Aoi T Kanagawa O Nakagawa M Okita K Yamanaka S Suppression of induced pluripotent stem cell generation by the p53-p21 pathway Nature 2009 460 1132 1135 19668191 10.1038/nature08235 PMC2917235

[B4] Kawamura T Suzuki J Wang YV Menendez S Morera LB Wahl GM Belmonte JC Linking the p53 tumour suppressor pathway to somatic cell reprogramming Nature 2009 460 1140 1144 19668186 10.1038/nature08311 PMC2735889

[B5] Utikal J Polo JM Stadtfeld M Maherali N Kulalert W Walsh RM Khalil A Rheinwald JG Hochedlinger K Immortalization eliminates a roadblock during cellular reprogramming into iPS cells Nature 2009 460 1145 1148 19668190 10.1038/nature08285 PMC3987892

[B6] Li H Collado M Villasante A Strati K Ortega S Canamero M Blasco MA Serrano M The Ink4/Arf locus is a barrier for iPS cell reprogramming Nature 2009 460 1136 1139 19668188 10.1038/nature08290 PMC3578184

[B7] Marion RM Strati K Li H Murga M Blanco R Ortega S Fernandez-Capetillo O Serrano M Blasco MA A p53-mediated DNA damage response limits reprogramming to ensure iPS cell genomic integrity Nature 2009 460 1149 1153 19668189 10.1038/nature08287 PMC3624089

[B8] Lin T Chao C Saito S Mazur SJ Murphy ME Appella E Xu Y P53 induces differentiation of mouse embryonic stem cells by suppressing Nanog expression Nat Cell Biol 2005 7 165 171 15619621 10.1038/ncb1211

[B9] Xu Y A new role for p53 in maintaining genetic stability in embryonic stem cells Cell Cycle 2005 4 3 363 4 15701975 10.4161/cc.4.3.1529

[B10] Mitsui K Tokuzawa Y Itoh H Segawa K Murakami M Takahashi K Maruyama M Maeda M Yamanaka S The homeoprotein Nanog is required for maintenance of pluripotency in mouse epiblast and ES cells Cell 2003 113 5 631 42 12787504 10.1016/s0092-8674(03)00393-3

[B11] Melino G De Laurenzi V Vousden KH p73: friend or foe in tumorigenesis Nat Rev Cancer 2002 2 605 615 12154353 10.1038/nrc861

[B12] Melino G Lu X Gasco M Crook T Knight RA Functional regulation of p73 and p63: development and cancer Trends Biochem Sci 2003 28 663 670 14659698 10.1016/j.tibs.2003.10.004

[B13] Grob TJ Novak U Maisse C Barcaroli D Lüthi AU Pirnia F Hügli B Graber HU De Laurenzi V Fey MF Melino G Tobler A Human delta Np73 regulates a dominant negative feedback loop for TAp73 and p53 Cell Death Differ 2001 8 12 1213 23 11753569 10.1038/sj.cdd.4400962

[B14] Chambers I Colby D Robertson M Nichols J Lee S Tweedie S Smith A Functional expression cloning of Nanog, a pluripotency sustaining factor in embryonic stem cells Cell 2003 113 5 643 55 12787505 10.1016/s0092-8674(03)00392-1

[B15] Loh YH Wu Q Chew JL Vega VB Zhang W Chen X Bourque G George J Leong B Liu J Wong KY Sung KW Lee CW Zhao XD Chiu KP Lipovich L Kuznetsov VA Robson P Stanton LW Wei CL Ruan Y Lim B Ng HH The Oct4 and Nanog transcription network regulates pluripotency in mouse embryonic stem cells Nat Genet 2006 38 431 440 16518401 10.1038/ng1760

[B16] Wang J Rao S Chu J Shen X Levasseur DN Theunissen TW Orkin SH A protein interaction network for pluripotency of embryonic stem cells Nature 2006 444 364 368 17093407 10.1038/nature05284

[B17] Theunissen TW Silva JC Switching on pluripotency: a perspective on the biological requirement of Nanog Philos Trans R Soc Lond B Biol Sci 2011 366 1575 2222 9 21727127 10.1098/rstb.2011.0003 PMC3130412

[B18] Grad I Hibaoui Y Jaconi M Chicha L Bergström-Tengzelius R Sailani MR Pelte NANOG priming before full reprogramming may generate germ cell tumours Eur Cell Mater 2011 22 258 74 22071697 10.22203/ecm.v022a20

[B19] Moon JH Kwon S Jun EK Kim A Whang KY Kim H Oh S Yoon BS You S Nanog-induced dedifferentiation of p53-deficient mouse astrocytes into brain cancer stem-like cells Biochem Biophys Res Commun 2011 412 1 175 81 Epub 2011 Jul 23 21810410 10.1016/j.bbrc.2011.07.070

[B20] Han JW Yoon YS Epigenetic landscape of pluripotent stem cells Antioxid Redox Signal 2012 in press doi:10.1089/ars.2011.4375 10.1089/ars.2011.4375 PMC3353817 22044221

[B21] Patterson M Chan DN Ha I Case D Cui Y Handel BV Mikkola HK Lowry WE Defining the nature of human pluripotent stem cell progeny Cell Res 2012 22 1 178 93 21844894 10.1038/cr.2011.133 PMC3351932

[B22] Uri Ben-David & Nissim Benvenisty The tumorigenicity of human embryonic and induced pluripotent stem cells Nat Rev Cancer 2011 11 268 277 21390058 10.1038/nrc3034

[B23] Lin T Ambasudhan R Yuan X Li W Hilcove S Abujarour R Lin X Hahm HS Hao E Hayek A Ding S A chemical platform for improved induction of human iPSCs Nat Methods 2009 6 805 808 19838168 10.1038/nmeth.1393 PMC3724527

[B24] Krizhanovsky V Lowe SW Stem cells: the promises and perils of p53 Nature 2009 460 1085 1086 19713919 10.1038/4601085a PMC2974062

